# Citrate metabolism and mitochondrial DNA signaling: cooperative regulation of the senescence-associated secretory phenotype

**DOI:** 10.1038/s41392-026-03089-2

**Published:** 2026-11-10

**Authors:** Geunhye Hong, Heeeun Yoon, Hae-Ock Lee, Jay H. Chung, Jeonghan Kim

**Affiliations:** 1 Department of Biochemistry, College of Medicine, The Catholic University of Korea, Seoul, South Korea; 2 Laboratory of Obesity and Aging Research, Cardiovascular Branch, National Heart Lung and Blood Institute, National Institutes of Health, Bethesda, MD USA; 3 Department of Microbiology, College of Medicine, The Catholic University of Korea, Seoul, Republic of Korea; 4 Department of Medical Sciences, Graduate School of The Catholic University of Korea, Seoul, South Korea

**Keywords:** Senescence, Inflammation

**Fig. 1:**
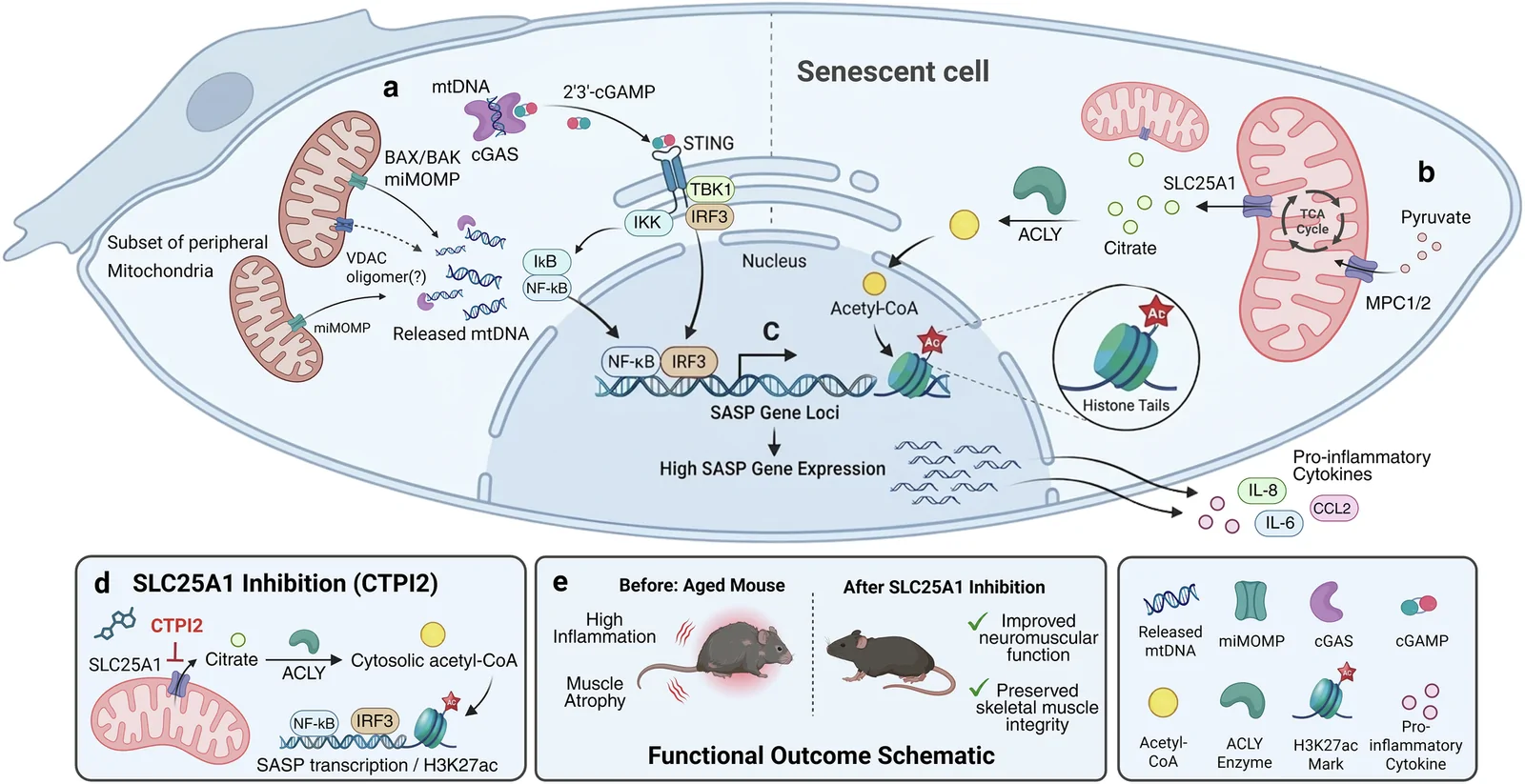
Mitochondrial citrate metabolism and mtDNA signaling converge to license SASP expression. **a** Minority mitochondrial permeabilization in senescent cells releases mtDNA into the cytosol, activating cGAS–STING signaling and downstream NF-κB/IRF3 inflammatory transcription factors. **b** Pyruvate import through MPC1/2 fuels mitochondrial citrate production; SLC25A1 exports citrate, which ACLY converts to nucleocytosolic acetyl-CoA. **c** Citrate-derived acetyl-CoA sustains H3K27ac and chromatin accessibility at SASP loci, enabling mtDNA-triggered transcription factors to drive robust SASP expression and cytokine secretion. **d** Pharmacological inhibition of SLC25A1 with CTPI2 reduces cytosolic acetyl-CoA and H3K27ac, suppressing SASP transcription without reversing senescence-associated growth arrest. **e** In aged mice, CTPI2 reduces SASP-associated inflammation and chromatin accessibility while preserving senescent-cell abundance, thereby delaying frailty and improving neuromuscular performance and skeletal muscle integrity. The figure was created by the authors using BioRender.com

In a recent study published in Nature, Martini et al. demonstrate that mitochondrial citrate export supplies acetyl-CoA for histone acetylation at senescence-associated secretory phenotype (SASP) loci, thereby licensing the transcriptional response to mitochondrial DNA (mtDNA)-driven innate immune signaling.^1^ Pharmacological inhibition of SLC25A1 suppresses SASP-associated chromatin accessibility and inflammation while preserving senescence-associated growth arrest, identifying a targetable metabolic–epigenetic checkpoint (Fig. 1).^1^

Cellular senescence couples durable cell-cycle arrest to a heterogeneous secretory program that can promote tissue repair and tumor suppression but, when sustained, contributes to chronic inflammation and age-associated dysfunction. Mitochondria have emerged as upstream regulators of this inflammatory output because mtDNA released through BAX/BAK-dependent minority mitochondrial outer membrane permeabilization (miMOMP) activates cyclic GMP–AMP synthase–stimulator of interferon genes (cGAS–STING) signaling and the SASP (Fig. 1a).^1,2^ However, activation of inflammatory transcription factors alone does not explain how SASP loci acquire and maintain a chromatin state permissive to sustained transcription. Martini et al. now identify mitochondrial metabolism as this previously unresolved regulatory layer.^1^

The authors first depleted mitochondria from senescent human fibroblasts through Parkin-mediated mitophagy. Mitochondrial depletion reduced histone H3 lysine 27 acetylation (H3K27ac) at SASP genes and suppressed inflammatory factors, including IL6 and CCL2, without compromising cell viability (Fig. 1b, c). Acetate supplementation, which replenishes acetyl-CoA through ACSS2, restored nuclear acetylation and partially rescued SASP expression.^1^ These findings extend to senescence the established principle that ATP-citrate lyase (ACLY) couples glucose-derived citrate to histone acetylation,^3^ revealing acetyl-CoA availability as a selective determinant of inflammatory chromatin.

The authors next delineated the metabolic pathway underlying this effect. The mitochondrial pyruvate carrier (MPC), SLC25A1, and ACLY were upregulated across multiple senescence models. Pharmacological or genetic disruption of MPC or SLC25A1 reduced SASP transcripts and secreted factors while preserving p16 and p21 expression, DNA-damage foci, and proliferative arrest; ACLY silencing similarly reduced H3K27ac and SASP expression (Fig. 1d).^1^ SLC25A1 thereby emerged as a central metabolic checkpoint. Inhibition with CTPI2 reduced cytosolic acetyl-CoA, global nuclear acetylation, and H3K27ac at SASP loci and abolished the incorporation of glucose-derived carbon into histone acetylation. By contrast, oxidative phosphorylation, ATP production, mitochondrial morphology, and reactive oxygen species remained largely unchanged, arguing against nonspecific mitochondrial dysfunction. These findings instead indicate that citrate export sustains a nucleocytosolic acetyl-CoA pool that maintains inflammatory regulatory elements in a transcriptionally competent state.

Reciprocal perturbation distinguished this metabolic input from mtDNA signaling. SLC25A1 inhibition suppressed SASP expression without reducing cytosolic mtDNA. Conversely, deletion of BAX and BAK1 decreased cytosolic mtDNA and SASP expression while leaving global histone acetylation and H3K27ac at SASP loci intact. STING inhibition likewise suppressed inflammatory genes without reducing histone acetylation.^1^ Collectively, these findings establish that mitochondrial citrate metabolism and mtDNA–cGAS–STING signaling are independently regulated yet functionally cooperative rather than components of a single linear cascade.

Reconstitution experiments further demonstrated this cooperation. Acetate increased H3K27ac independently of STING activity, whereas mtDNA transfection failed to increase histone acetylation in mitochondria-depleted senescent cells. Acetate or mtDNA alone induced only a subset of the inflammatory program, whereas their combination elicited substantially greater SASP activation.^1^ These data support a two-input model in which mtDNA provides the inflammatory trigger, while citrate-derived acetyl-CoA provides the epigenetic license required for robust transcriptional activation.

The dissociation of growth arrest from inflammatory output has important therapeutic implications. In aged mice, CTPI2 delayed frailty, improved coordination and strength, preserved skeletal muscle integrity, and reduced circulating and tissue inflammatory factors (Fig. 1e). Single-nucleus multiome profiling revealed reduced SASP transcription and chromatin accessibility in senescent-like muscle nuclei without a measurable decrease in their abundance. In the liver, nuclear acetylation was reduced in p16- and/or p21-positive, PCNA-negative cells without reducing the number of these cells.^1^ SLC25A1 inhibition may therefore attenuate the detrimental secretory state without requiring elimination of senescent cells. However, bone microarchitecture was not improved, and some hepatic responses differed by sex, underscoring the need to define tissue-specific efficacy, pharmacodynamic windows, and long-term safety.

The study also sharpens an important mechanistic question: which mitochondrial pore supplies the mtDNA “key” under distinct senescence-associated stresses? BAX/BAK-dependent miMOMP is an established mechanism in senescent cells, enabling mtDNA release without committing the entire cell to apoptosis.^2,4^ VDAC-dependent mtDNA release has also been reported in senescent cells. VDAC1/3 can form oligomeric pores in viable cells that release mtDNA fragments, providing a mechanistically distinct route from BAX/BAK-dependent miMOMP.^4^

These observations suggest that VDAC-mediated permeabilization may represent a senescence-associated route of mtDNA release that functions alongside BAX/BAK-dependent miMOMP or is preferentially engaged under distinct conditions. Depending on the senescence-associated stress context, progression beyond miMOMP to widespread apoptotic MOMP may activate caspase-3, which can cleave and inactivate cGAS and IRF3, thereby attenuating downstream signaling.^2,4,5^ A central unresolved question is how BAX/BAK-dependent miMOMP is coordinated with the VDAC oligomeric-pore pathway, whether the relative contributions of these routes vary with the senescence-inducing stimulus and cellular context, and whether the route of mtDNA release determines the balance between intracellular and extracellular signaling. Defining the interplay between these pore-forming pathways may reveal how distinct mtDNA-release mechanisms shape inflammatory signaling networks and uncover new therapeutic opportunities to suppress senescence-associated inflammation.

By linking mitochondrial citrate export to inflammatory chromatin regulation, Martini et al. recast the SASP as the product of coordinated mitochondrial signaling and metabolism. Targeting this metabolic license may provide a strategy to restrain age-associated inflammation while preserving potentially beneficial functions of cellular senescence.
